# The genetic aetiology of cannabis use: from twin models to genome-wide association studies and beyond

**DOI:** 10.1038/s41398-022-02215-2

**Published:** 2022-11-21

**Authors:** Karin J. H. Verweij, Jacqueline M. Vink, Abdel Abdellaoui, Nathan A. Gillespie, Eske M. Derks, Jorien L. Treur

**Affiliations:** 1 Department of Psychiatry, Amsterdam UMC, University of Amsterdam, Meibergdreef 5, 1105 AZ Amsterdam, The Netherlands; 2 Behavioural Science Institute, Radboud University Nijmegen, Thomas van Aquinostraat 4, 6525 GD Nijmegen, The Netherlands; 3 Virginia Institute for Psychiatric and Behavior Genetics, Virginia Commonwealth University, 800 East Leigh St, Suite 100, Richmond, VA 23219 USA; 4 Translational Neurogenomics, QIMR Berghofer Medical Research Institute, 300 Herston Road, Herston, QLD 4006 Australia

**Keywords:** Genetics, Neuroscience

## Abstract

Cannabis is among the most widely consumed psychoactive substances worldwide. Individual differences in cannabis use phenotypes can partly be explained by genetic differences. Technical and methodological advances have increased our understanding of the genetic aetiology of cannabis use. This narrative review discusses the genetic literature on cannabis use, covering twin, linkage, and candidate-gene studies, and the more recent genome-wide association studies (GWASs), as well as the interplay between genetic and environmental factors. Not only do we focus on the insights that these methods have provided on the genetic aetiology of cannabis use, but also on how they have helped to clarify the relationship between cannabis use and co-occurring traits, such as the use of other substances and mental health disorders. Twin studies have shown that cannabis use is moderately heritable, with higher heritability estimates for more severe phases of use. Linkage and candidate-gene studies have been largely unsuccessful, while GWASs so far only explain a small portion of the heritability. Dozens of genetic variants predictive of cannabis use have been identified, located in genes such as *CADM2*, *FOXP2*, and *CHRNA2*. Studies that applied multivariate methods (twin models, genetic correlation analysis, polygenic score analysis, genomic structural equation modelling, Mendelian randomisation) indicate that there is considerable genetic overlap between cannabis use and other traits (especially other substances and externalising disorders) and some evidence for causal relationships (most convincingly for schizophrenia). We end our review by discussing implications of these findings and suggestions for future work.

## Introduction

Cannabis is among the most widely consumed psychoactive substances worldwide. An estimated 4% of the world population aged 15 to 64 used cannabis at least once in 2019 [1]. While prevalences vary highly between countries, the overall European Union lifetime use prevalence is estimated to be 27.2% [2]. People mainly use cannabis to experience a psychoactive induced ‘high’ characterised by mild euphoria, relaxation, and perceptual and cognitive alterations [3]. These responses are likely related to the endogenous endocannabinoid system, given that Δ-9-tetrahydrocannabinol (THC) binds to cannabinoid receptors in different brain areas. Besides THC, an important component of cannabis is cannabidiol (CBD). By itself, CBD is not intoxicating (at typical doses) and has a much lower risk of adverse effects compared to THC [4]. This is confirmed by studies showing that cannabis with an elevated THC to CBD ratio is more damaging [5].

Indeed, a large body of research has demonstrated adverse effects linked to cannabis use. For example, cannabis use is associated with accidents, lower cognition and motivation, and suicide attempts and regular use has been related to various physical and psychological problems [5–8]. Regular use can also lead to addiction; in many countries cannabis is among the most common primary reasons for entering drug-related treatment [1] and cannabis use often precedes other drug use [9–11]. Problems related to cannabis use can in turn interfere with family, school, and work obligations [12]. Public health costs, law enforcement, and loss of work potential because of cannabis use are an economic drain on society [13]. In contrast, there may also be positive health benefits. There is some evidence that, by itself, CBD has antioxidant, anti-inflammatory, and neuroprotective properties [5]. Cannabinoid-based drugs are used to treat a range of medical conditions, including neurological disorders, psychiatric disorders, and pain [4, 14, 15]. While few serious side-effects have been reported, additional safety data are needed from more (and larger) clinical trials. In addition, it is important to note that non-medicinal CBD products (sold online or from health food retailers) lack quality standards and are not recommended for medicinal purposes [4].

In light of the prevalence and adverse effects, for prevention, intervention and harm reduction efforts to be effective, it is important to understand why some individuals initiate cannabis use while others do not, and why a small subset progresses to regular user or develop a cannabis use disorder (CUD). In addition to environmental factors known to increase use (e.g. peer substance use, lower socio-economic status, poor neighbourhood characteristics, inadequate parental monitoring, high drug availability, and stressful life events [16–20]), risk of cannabis use runs also in families. A substantial part of the variability in cannabis use is due to genetic differences. This review provides an overview of current knowledge of the genetics of cannabis use, covering early twin studies to genome-wide association studies (GWASs) and post-GWAS analyses. When presenting results, we will refer to various indices of cannabis use, including initiation, frequency of use, and CUD which also be operationalised differently per study (Box 1 provides an overview of phenotypic definitions).

Box 1 Definitions of the cannabis use variables Throughout the literature various measures and descriptions have been used as indices of cannabis use. In this review we use the term **cannabis use** as a generic, inclusive term to describe all types of cannabis use behaviours. Definitions of specific phenotypes are:

**Cannabis initiation;** self-reported information on whether or not a person has ever used cannabis in their lifetime, sometimes referred to as ‘lifetime cannabis use’.

**Frequency of cannabis use;** a measure of the number of times that cannabis is used during a specified period (e.g. one week or one month).

**Quantity of cannabis use;** a measure of the amount of cannabis that is used during a specified period (e.g. a single occasion, or during one week or one month).

**Cannabis use disorder (CUD);** umbrella term for a clinical diagnosis, in the DSM subdivided into cannabis dependence and cannabis abuse.

**Cannabis abuse;** a diagnosis of cannabis abuse, based on DSM criteria or ICD codes. Cannabis abuse can be defined as a maladaptive pattern of substance use manifested by recurrent and significant adverse consequences related to the repeated use.

**Cannabis dependence;** a diagnosis of cannabis dependence, based on DSM criteria or ICD codes. Cannabis dependence is the more severe form of cannabis use disorder can can be defined as “a cluster of cognitive, behavioural, and physiological symptoms indicating the individual continues use despite significant substance-related problems. There is a pattern of repeated self-administration that usually results in tolerance, withdrawal, and compulsive drug-seeking behaviour.

**Problematic cannabis use;** term used to refer to use of cannabis to an extent to which it leads to problems (symptoms of abuse/dependence or a full diagnosis of abuse/dependence).

**Age at first cannabis use;** self-reported information on the age at which a person used cannabis for the first time.

## Twin studies

Before detailed information on the DNA sequence of the human genome was available, scientists were limited to studies using inferred genetic relatedness to explore the influence of genetic factors on cannabis use. Such studies relied on family, adoption, and twin designs. Family studies cannot distinguish between genetic and family environmental influences, and only few adoption studies were performed of cannabis use. A longitudinal adoption study showed that genetic influences on cannabis initiation were important at an early age (13–14 years old), but less so at age 17 and 18 [21].

Twin studies have proven more valuable because they typically used larger samples than adoption studies and they can differentiate between shared environmental and genetic influences. In twin studies, the resemblance between monozygotic twin pairs (who share all their DNA) is compared to that of dizygotic twin pairs (who share on average 50% of their segregated genes) [22]. If monozygotic twins resemble each other more than dizygotic twins on a certain trait, for example cannabis use, this is an indication that this trait is partly influenced by genetic difference between people. By applying sophisticated statistical models to twin data, it is possible to estimate what proportion of individual differences is due to genetic differences between people (heritability), shared (or family) environmental, and non-shared (or unique) environmental influences (see [23, 24]). Decades of twin studies have revealed that virtually every physical, behavioural, cognitive, and disease trait is heritable [25]. It is important to emphasise that heritability does not represent a fixed estimate nor does it describe individual levels of personal risk. Estimates of genetic and environmental variation are population estimates used to describe the sources of individual differences within a sample. When a trait or disorders is heritable, this does not mean that people’s outcomes are determined at conception; heritability does not equal genetic determinism. Instead, whether someone develops a certain disease or addiction depends on a complex interplay between genetic vulnerability and many environmental factors.

The heritability of various cannabis use phenotypes has been estimated in twin studies, most of which focussed on cannabis initiation or indices of CUD. A meta-analysis of these twin studies in 2010 [26] presented meta-analytic heritability estimates of 48% for females and 51% for males for cannabis initiation, and 51% for females and 59% for males for problematic cannabis use. In addition, Agrawal et al. [27] estimated a heritability of 35% and 27% for positive and negative subjective initial reactions to cannabis intake, Hines et al. [28] estimated that the opportunity to use cannabis was 64% heritable, and frequency of use 74%, and Minică et al. [29] estimated that age at first cannabis use was 38% heritable. In general, the relative genetic contribution is lower for the initiation of cannabis use compared to more severe stages of use such as problematic use, while for shared environmental influences, it was the other way around. Possibly, the initial stages of cannabis use are more sensitive to environmental factors, such as drug availability, peer influences, parental monitoring, and parental attitudes towards drug use, whereas the likelihood of progression to problematic use is more influenced by biological factors such as people’s physical response to THC intake. The pattern of higher heritability and lower family environmental influences for more severe phases of cannabis use has also been found for other substances [30, 31].

With multivariate twin methods [23], it is possible to estimate how much the genetic influences on one trait overlap with those underlying other traits. Multivariate twin studies have revealed that large portions of genetic factors in cannabis use initiation and problematic use are shared [32]. Correlations between measures of cannabis initiation, regular use, and problematic use suggest a single liability [33, 34], explained by common genes and environments [31, 32]. Similar patterns in terms of common genetic and environmental on different stages of use have also been observed for other substance [35, 36].

Multivariate twin studies have also explored to what extent genetic and environmental influences are shared across use of different substances. One study found that a common factor influenced by genetic factors, and family and non-family environmental influences underpins comorbid cannabis, sedative, stimulant, opioid, and psychedelic misuse [37]. Another study found that comorbid substance misuse (including cannabis, cocaine, hallucinogens, sedatives, stimulants, and opiates) is largely explained by overlapping genetic and shared environmental influences [38]. The same study also suggest that random environments determine how individuals choose to use a particular substance. A third study found that comorbid cannabis, cocaine, alcohol, caffeine, and nicotine misuse was best explained by two highly correlated genetic factors - one predisposing to cannabis and cocaine, the other to alcohol, caffeine, and nicotine misuse [39].

Overall, twin studies have demonstrated substantial overlap in genetic factors influencing earlier (experimental/regular use) and later (CUD) stages of cannabis use, and significant genetic overlap between use of cannabis and other substances. This general genetic vulnerability to substance use could be part of a much broader spectrum of personality characteristics or externalising psychopathology, characterised by substance use as well as conduct disorder, antisocial personality disorder, and other correlated traits [40–45].

## Gene-finding studies

With the arrival of affordable DNA genotyping, the focus of behavioural genetics research shifted from family and twin studies to designs such as linkage analysis, candidate-gene studies, and GWASs, which rely on measured genotypes. Linkage analyses test for co-inheritance of genetic markers and traits within families. The segregation of a genetic marker within families is compared with the segregation of the trait in the family members. Downsides of this approach are that the analysis requires pedigree data and that linkage peaks only provide a rough indication of the implicated genomic region. In genome-wide linkage studies of cannabis use, most linkage peaks did not meet significance, and nearly all failed to replicate [46–51]. Ehlers et al. [48] found genome-wide significant linkage peaks for symptoms of cannabis dependence on chromosome 16 and 19, and in another study [49] on chromosomes 1, 3, 6, 7, and 9 for craving and cannabis symptoms. Hopfer et al. [50] reported suggestive evidence for linkage peaks for cannabis dependence symptoms on chromosome 3 and 9 in an adolescent sample. Han et al. [51] found a (non-significant) linkage peak at chromosome 8 for cannabis dependence; they then performed an association analysis under this peak, and found a significant and replicable association between variants in *NRG1* and cannabis dependence. Non-significant peaks were reported on chromosome 14 for cannabis dependence symptoms [46], on chromosome 18 for cannabis frequency of use and initiation and chromosome 19 for early onset of cannabis use [47], and on chromosome 1 and 4 for cannabis problems [52]. The latter peak was in the region of the gamma-aminobutyric acid type A gene cluster, which includes *GABRA2* that had previously been implicated in drug use disorders [53, 54].

Around the same time researchers also turned to candidate-gene studies, a hypothesis-driven method designed to tests for a correlation between a phenotype and a gene that is hypothesised to relate to this phenotype. For cannabis use these studies focused initially on variants in the cannabinoid receptor (*CNR1*) gene, located at chromosome 6. *CNR1* is densely expressed in the central nervous system, notably in brain circuits thought to be important for reward and mnemonic processes related to substance misuse [55]. *CNR1* was among the strongest candidate genes for cannabis use because it was known to be activated not only by endocannabinoids, but also plant phytocannabinoids such as THC, and synthetic analogs of THC. Using the candidate-gene approach, Hopfer et al. [56] and Agrawal et al. [57] found a significant association between the *CNR1* gene and (symptoms of) cannabis dependence, but others could not replicate this association [58, 59]. In a meta-analysis, Benyamina et al. [60] showed a small but significant effect for the *CNR1* AAT polymorphism on measures of substance dependence that included cannabis. Candidate-gene studies have also reported associations between cannabis use phenotypes and *GABRA2* [53, 61], *FAAH* [62–64], and *ABCB1* [65]. However, these associations largely failed to replicate [66–68]. For a comprehensive overview of candidate-gene studies for CUDs, see [69].

Overall, linkage and candidate-gene association studies were largely unsuccessful at identifying replicable genes. This failure is likely attributable to variation in research designs and phenotyping, lack of power, and publication bias [70]. Fortunately, technological advances permitted genome-wide analysis of genetic variants associated with complex traits, using GWASs. GWASs use genetic markers (typically single nucleotide polymorphisms (SNPs)) spanning the entire genome to systematically test for association with a trait. This approach has become a widely adopted method of identifying genetic associations.

The first cannabis GWASs, focussed on initiation [71, 72], dependence [73], and age at initiation [72], comprised small sample sizes and failed to identify genome-wide significant genetic loci. To increase power, large-scale collaborative efforts were undertaken. In 2012, the *International Cannabis Consortium* (ICC) was established with the aim of combining data from multiple cohorts to identify genetic variants associated with cannabis use. To date, the ICC has published three GWAS meta-analyses. The first [74] investigated cannabis initiation and involved a meta-analysis of 13 cohorts (*N* = 32,330, plus four replication samples (*N* = 5,627)). Although no individual SNP reached genome-wide significance, subsequent gene-based tests of association identified four genes significantly associated with cannabis initiation: *NCAM1*, *CADM2*, *SCOC*, and *KCNT2*. In a more recent ICC report [75], where the meta-analytic sample for cannabis initiation was increased to ~184,000 individuals, eight independent genome-wide significant SNPs in six regions were identified, as well as 35 significant genes in a gene-based tests of association. The third ICC GWAS report investigated age at onset of cannabis use (*N* = 24,953 individuals [29]), and identified a genome-wide significant association with SNPs in the *ATP2C2* gene.

In 2016, Sherva et al. [76] identified the first genome-wide significant associations for cannabis dependence. The performed a GWAS for cannabis dependence criterion count in three substance dependence cohorts (*N* = 14,754 African American and European American participants; 18–36% cases). Three independent genome-wide significant SNPs were identified, two specific to African American participants (one in *RP11-206M11.7* and one 12.4 kb upstream from the S100B gene) and one in the combined sample (in the *CSMD1* gene). Two additional meta-analytic efforts for cannabis use disorder have been undertaken by (i) the Initiative for Integrative Psychiatric Research (iPsych) and deCODE genetics [77] and (ii) the Psychiatric Genetics Consortium—Substance Use Disorder (PGC-SUD) workgroup [78]. Demontis et al. [77] performed a GWAS for CUD with a discovery sample of 2,387 cases and almost 50,000 controls (plus a replication sample of 5,501 cases and ~300,000 controls). They identified one genome-wide significant risk locus for CUD, a SNP that is a strong marker for *CHRNA2* expression. More recently, The PGC-SUD GWAS meta-analysis study based on 20,916 cases and 363,116 controls [78] identified two genome-wide significant loci: one novel locus in the *FOXP2* gene, and the previously identified locus near *CHRNA2* (and *EPHX2*). A systematic review of all cannabis use GWASs can be found elsewhere [79].

**Fig. 1:**
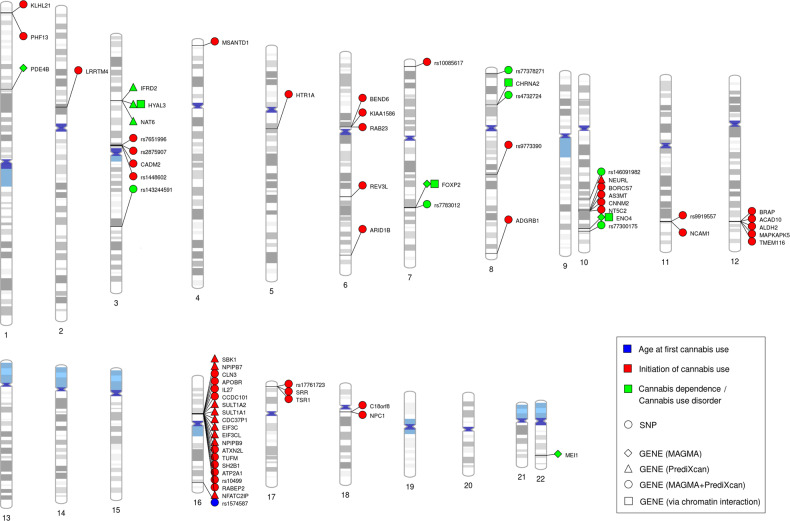
Genomic locations of the identified genome-wide significant SNPs and genes. Illustration of the genomic locations of the genome-wide significant SNPs and genes for cannabis use phenotypes as identified in genome-wide association studies.

Whole genome sequencing (WGS) allows for more comprehensive association analysis than microarray-based GWASs, with the potential to identify rarer genetic variants. Gizer et al. [80] applied low-pass WGS to identify low frequency variants involved in cannabis dependence across two cohorts: a Native American tribal community and a family-based sample of primarily European ancestry. Their set-based analysis yielded two significant regions: a protein-coding region, *C1orf110*, and a regulatory region within the *MEF2B* gene. An overview of significant SNP and gene-based associations from the GWAS and WGS reports can be found in Table 1 and Fig. 1.

**Table 1:** Functional annotation of genetic findings provides insight into the molecular mechanisms underlying statistical associations.

Phenotype	Study	Lead SNP	Chr	Position (base pair)	A1	A2	Freq A1	Effect size^a^	SE	*p*-value	*N*	Nearby genes/transcripts	Functional annotation	
													Method	Annotated gene(s)
Cannabis initiation	Pasman et al. [75]	rs2875907	3	85518580	A	G	0.352	0.070	0.009	9.38e−17	181,675	*CADM2*	MAGMA	35 genes (*NCAM1* and *CADM2* among top findings)
		rs1448602	3	85780454	A	G	0.756	−0.062	0.010	6.55e−11	184,765	*CADM2*	TWAS (PrediXcan)	21 genes (*CADM2* among top findings)
		rs7651996	3	85057349	T	G	0.477	0.049	0.008	2.37e−09	184,765	*CADM2*		
		rs10085617	7	3634711	A	T	0.416	0.046	0.008	2.93e−08	184,765	*SDK1*		
		rs9773390	8	81565692	T	C	0.933	−0.171	0.029	5.66e−09	44,595	*ZNF704*		
		rs9919557	11	112877408	T	C	0.614	−0.055	0.009	9.94e−11	180,428	*NCAM1*		
		rs10499	16	28915527	A	G	0.651	0.053	0.009	1.13e−09	179,767	*RABEP2; ATP2A1*		
		rs17761723	17	2107090	T	C	0.346	0.047	0.009	3.24e−08	184,765	*SMG6*		
Age at first cannabis use	Minică et al. [29]	rs1574587	16	84453056	T	C	0.142	0.090	0.016	4.0e−09	24,953	*ATP2C2*	GATES gene-based analysis	*ATP2C2*
Cannabis dependence^a^	Agrawal et al. [96]	rs77300175	10	120633376	T	C	0.050^b^	0.530	0.09	1.3e−08	2080 cases 6435 controls	12 genes within regulatory domain	Genomic and epigenomic annotation of rs1409568	Located within active enhancer; enrichment of H3K27ac marks; predicted to bear active enhancer marks in brain-derived tissues; changes of CpG methylation of *TIAL1;* Association with hippocampal volume in college students
Cannabis Dependence (criterion count)	Sherva et al. [76]	rs143244591	3	149013935	G	A	0.96	0.54	NA	4.32 × 10^−10^	6000 African American and 8754 European American participants	RP11-206M11.7	NA	NA
		rs146091982	10	95659958	A	G	0.95	0.54	NA	1.33 × 10^−9^		*SLC35G1*		
		rs77378271	8	3073489	A	G	0.95	0.29	NA	2.13 × 10^−8^		*CSMD1*		
Cannabis use disorder	Johnson et al. [78]	rs7783012	7	114116881	A	G	0.569^b^	0.104	0.02	1·84 × 10^−9^	17,068 cases, 357,219 controls	*FOXP2*	eQTL analysis	*FOXP2*
		rs4732724	8	27432062	C	G	0.214^b^	−0.117	0.02	6·46 × 10^−9^	Half of the sample	CHRNA2; EPHX2	eQTL analysis	*CHRNA2; EPHX2; CCDC25; CLU; STMN4*
													MAGMA^c^	*FOXP2; PDE4B; ENO4*
													TWAS (PrediXcan)	*NAT6; HYAL3; IFRD2*

*SNP* single nucleotide polymorphism, *Chr* chromosome.

^c^Johnson et al. also report H-MAGMA results [146], but have been excluded here as these results may include an excessive number of false positive findings due to incorrect specification of the null distribution in MAGMA; a problem that has been resolved in the latest MAGMA version.

^a^For this GWAS, the control sample was selected to have used cannabis during their life (i.e. those who had never used cannabis were excluded from analyses).

^b^Note that these A1 allele frequencies were obtained from https://www.ncbi.nlm.nih.gov/snp/ (European estimates), whereas for the other SNPs the A1 allele frequencies were reported in the original GWASs, as obtained from the included samples.

## Revealing molecular mechanisms using functional annotation analyses

GWASs alone cannot inform the cascade of biological changes linking SNPs to cannabis use. This can, however, be addressed using gene-expression analyses via analysis of expression quantitative trait loci (eQTLs) or SNPs regulating gene-expression. Because gene-expression plays a critical role in human diseases [81], and because eQTLs regulate gene-expression, they likely provide a direct link between GWAS results and gene-expression studies [82]. Furthermore, eQTL analysis can discern transcriptome adaptations, while eQTLs in transcription factor binding sites, splice sites, and regulatory regions can reveal mechanisms by which genetic variants contribute to cannabis use [83]. Since most variants reside outside protein-coding regions, the influence of eQTLs on cell functioning likely involves subtle modification of gene transcription and translation [84]. By assessing eQTLs in linkage disequilibrium with SNPs associated with cannabis use, can we begin to explain their function.

Many of the genetic variants associated with cannabis use are located in non-protein-coding regions. Therefore, functional annotation analyses are required to elucidate downstream biological consequences underlying these genetic associations. Several methods have been developed for the biological interrogation of genetic associations [85]. The majority are based on the premise that associated SNPs influence disease risk by their influence on an intermediate molecular trait (known as a quantitative trait locus), such as gene expression, protein expression, exon splicing, or DNA methylation.

Functional annotation analyses of cannabis use are relatively sparse as only a handful studies have revealed significant genome-wide associations. However, the results reveal interesting leads to putative causal genes (Table 1). Multiple studies have explored if associated genetic variants regulate gene expression by browsing databases of expression Quantitative Trait Loci (eQTLs). Demontis et al. [77] found that genetic variants linked to CUD are eQTLs for *CHRNA2*, a nicotinic acetylcholine receptor gene. This finding was confirmed in the larger PGC-SUD GWAS meta-analysis [78] (including the Demontis sample). Given the association between *CHRNA2* and cigarette smoking [86], Demontis et al. [77] explored whether the association between this gene and CUD was due to smoking as a confounding factor. Their results suggest that the signal is primarily driven by CUD.

Transcriptome-wide association study (TWAS) combines eQTL information across SNPs and tests the association between imputed levels of gene expression and disease risk to prioritise risk genes in a tissue-specific manner [87, 88]. Using the TWAS approach, Demontis et al. [77] found an association between CUD and *CHRNA2* expression in the cerebellum, whereas Johnson et al. [78] found significant associations between CUD and expression levels for *NAT6* (amygdala, cortex, frontal cortex), *HYAL3* (multiple brain tissues), and *IFRD2* (cerebellum). Significant associations were also reported between CUD and expression in *NAT6*, *HYAL3*, *SHTN1*, and *FOXP2* in other tissues such as whole blood and adipose [78], highlighting the potential for non-invasive predictive bio-markers of CUD.

A TWAS of cannabis initiation [75] revealed 21 genes of which imputed expression levels are associated with initiation. The top association was found for *CADM2*; genetic variants associated with increased liability to initiate cannabis use are predicted to upregulate expression levels in eight non-brain tissues, including whole blood. *CADM2* has been found to be associated with risk-taking, impulsivity, several measures of substance use, risky sexual behaviour, and self-control [89–95], suggesting that the association with cannabis use is part of a spectrum of externalising traits.

Agrawal et al. [96] conducted an extensive exploration of the molecular mechanisms underlying the association between rs1409568 and cannabis dependence. Based on its regulatory effects, this SNP was identified as the most plausible functional candidate within a locus at chromosome 10. The SNP appears to be located within an active enhancer and was predicted to bear active enhancer marks in several brain-derived tissues (e.g. dorsolateral prefrontal cortex). The risk increasing C allele is associated with reduced binding of several transcription factors. There was some support for this SNP to be associated with CpG methylation of *TIAL1*, with lower methylation scores in C allele carriers. Finally, the C allele of rs1409568 was also associated with a modest increase in right hippocampal volume (2.13%) in a sample of college students of whom very few met criteria for cannabis dependence. Of note, the counterintuitive finding of *increased* rather than *decreased* volumes was replicated in the phenotypic analysis.

## Post-GWAS approaches

As with nearly all complex traits, GWAS has likewise revealed that cannabis use is a highly polygenic behaviour whereby individual differences are explained by many genetic variants each with very small effects. These tiny individual differences combined explain considerable amounts of genetic variation, but current GWASs capture only a fraction of the estimated heritability reported by twin studies. For instance, SNP-based heritability estimates are 11% for cannabis initiation [75], 3.6% for age at initiation [29], and between 6.7 and 12.1% (depending on the estimated population prevalence) for cannabis dependence [78]. The discrepancy in heritability reported by twin studies and GWASs is referred to as ‘missing heritability’, and is a known phenomenon in complex traits [97]. Among the various explanations proposed, missing heritability may arise from rare variants not captured by SNP arrays used in GWASs, or the poor ability of current genotyping arrays to capture structural variants. It is also possible that there may be interplay between genes and the environment not captured with the current GWAS design. As study sample sizes and genomic coverage increase, the expectation is for SNP-heritability to increase. Despite the small individual effect sizes and low SNP-heritabilities, summary-level data from GWASs—containing the association estimates of each genetic variant with the outcome variable—can be used for a range of useful secondary analyses. It is anticipated that this research will improve our understanding of the genetic architecture of cannabis use, and will help elucidate the nature of the relationships between cannabis use and comorbid complex traits including mental health outcomes.

## Polygenic score analyses

Polygenic scores (PGSs) are predictors of the genetic liability of an individual to a disease or trait, and can be calculated by summing an individual’s ‘risk’ alleles for a certain phenotype weighted by the allele effect size, which are typically derived from effect estimates from large-scale GWASs. While PGSs only capture a small part of the genetic contribution to a trait, the validity of PGSs to predict complex psychiatric behaviours has been well demonstrated for many traits (e.g. [98–100]). Since the publications of the large-scale GWASs on cannabis initiation, age at initiation, and CUD, a number of studies have used the summary statistics to create PGSs in independent samples to predict observed cannabis use, other substance use, or correlated phenotypes. Several studies have found that cannabis PGSs significantly predict cannabis use phenotypes [77, 101–105] and mental health problems including depression and self-harm [103, 106], whereas other PGS analyses have not yielded significant results [105, 107, 108]. With larger samples we can determine if such discrepancies stem from lack of statistical power.

## Using GWAS results to examine genetic correlations between traits

**Fig. 2:**
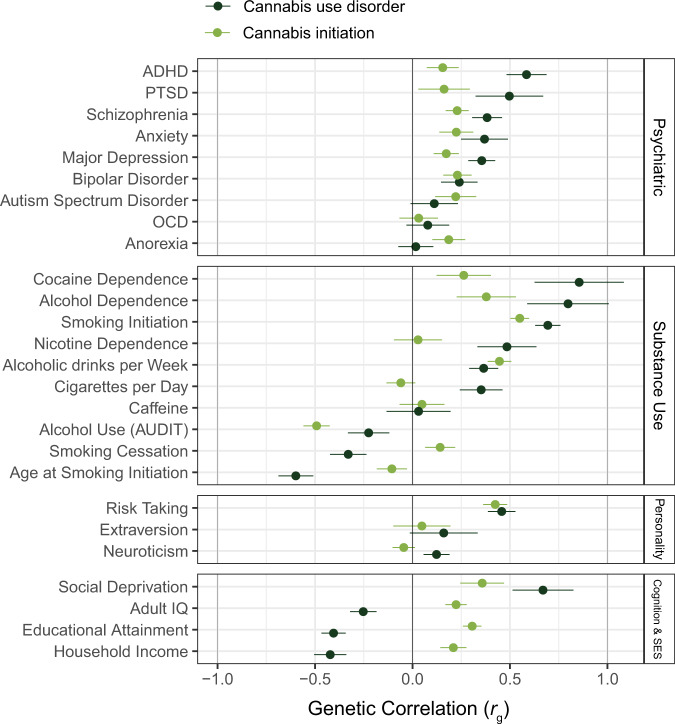
Genetic correlations of cannabis initiation and cannabis use disorder with behavioral and mental health outcomes. The genetic correlations were computed with LD Score regression and the GWAS summary statistics of the GWASs on these traits (see Supplementary Table 1 for references and sample sizes).

The introduction of affordable genotyping has meant that twin-based findings regarding sources of comorbidity of cannabis use with use of other substances or correlated traits can now be tested using measured genotypes. To assess shared genetic risks, linkage disequilibrium score regression (LDSR) can be used to compute genetic correlations between traits using summary-level GWAS data [109]. Such genetic correlation reflects the degree to which effects of genetic variants across the genome on one trait correlate with those on a second trait. Genetic correlations between cannabis use and various relevant other traits are shown in Fig. 2. Strong genetic correlations are found between cannabis use and other substance use. Cannabis initiation is strongly correlated with smoking initiation, whereas CUD is strongly correlated with dependency, e.g. alcohol dependence and cocaine dependence. This suggests a common genetic liability for initiating an addictive substance, and a partly distinct genetic liability for progressing from initiation to heavier use. Initiating substance use is most likely influenced by genetic factors that relate to externalising traits such as impulsivity. In line with this risk-taking and ADHD show some of the strongest genetic correlations with cannabis use. Cannabis use is also considerably correlated with major mental health disorders, e.g. major depressive disorder, schizophrenia, and bipolar disorder. Overall, these patterns imply that cannabis use has a considerable shared genetic aetiology with mental health problems. Note that while for cannabis initiation there are positive genetic correlations with intelligence, educational attainment, and income, these genetic correlations are negative for CUD.

By itself, a genetic correlation does not inform about the mechanisms underlying the association. Genomic structural equation modelling (SEM [110]) addresses this gap by providing insights into the nature of genetic associations. Genomic SEM is an extension of LDSR used to estimate genetic covariance between multiple traits using GWAS data. By constructing latent variables, different types of models can be built and (sub)models can be compared to test which has the superior fit. A number of studies have used genomic SEM to investigate relationships between cannabis use and other traits by modelling a latent genetic factor structure. One study included different substance use traits, and identified a unidimensional addiction risk factor, in which cannabis use (together with opioid use disorder) demonstrated the largest loadings [111]. Two other studies looked at mental health variables more broadly, and both found that cannabis dependence is part of a larger (externalising) factor comprised of, among others, alcohol dependence, nicotine dependence, and ADHD [112, 113].

## Using GWAS results for causal inference

While a genetic correlation could arise due to a shared genetic liability between trait X and trait Y (‘*horizontal pleiotropy’*), this is not the only possible explanation. If there are causal relationships, such that X *causes* Y, or Y *causes* X, this would also lead to genetic correlations (‘*vertical pleiotropy’*) [114]. For example, if cannabis use causes schizophrenia, then genes underlying cannabis use should be indirectly associated with schizophrenia. Resolving the direction of causation may help improve preventive efforts. A genetic method that aims to infer causality using summary-level GWAS data is Mendelian randomisation (MR). To conduct an MR study, genetic variants that are strongly and reliably predictive of the proposed risk factor are typically required. Usually, this is achieved by selecting genetic variants that are genome-wide significantly (*p* < 5E-08) associated with the proposed risk factor in a well-powered GWAS. In some cases variants are selected based on a higher *p*-value threshold (e.g. *p* < 1E−07 or *p* < 1E−05). This is generally done when there is a lack of available genome-wide significant variants (note that this practice can lead to weak instrument bias). The selected variants are then employed as instrumental variables, or ‘proxies’, to test causal effects on an outcome. MR can be compared to a randomised clinical trial (RCT) in the sense that experimental randomisation into an ‘exposed’ and an ‘unexposed’ group is mimicked by the random assortment of a set of genetic variants. Genetic differences on these variants should not be (strongly) associated with confounders, which reduces bias [115].

There are important assumptions that need to be fulfilled to justify a causal interpretation of an MR analysis. The three main assumptions are that the genetic instrument must (1) be robustly associated with the exposure variable, (2), not be associated with any confounding variables, and (3) not influence the outcome through another path than through the exposure. Additional assumptions depending on the exact MR design are discussed elsewhere [116]. In general, it is preferable to use genetic instruments for which there is a (relatively) good understanding of how genetic variation leads to individual differences in the trait. For cannabis use, knowledge of biological pathways is limited and as mentioned before, there is evidence that the genetic variants involved are highly pleiotropic. This should be taken into account when judging evidence from MR studies looking at cannabis use. An important strength of MR is that a wide range of sophisticated sensitivity methods is available to assess the robustness of a causal finding.

MR studies have so far focused on two topics, the first being the relationship between cannabis use and the use of other substances. Three studies attempted to elucidate causal pathways of cannabis use with smoking, caffeine consumption, alcohol use, and other drug use (cocaine and opioid dependence), specifically trying to establish whether there is some kind of gateway mechanism. The first study (N = 38,181 to 112,117) found no clear evidence for causal relationships [117]. The second study (*N* = 25,153 to 207,726) found no evidence for causality except for one relationship: smoking initiation leading to higher caffeine intake [118]. The most recent MR study (*N* = 1749 to 1,232,091), which was also the most extensive with regards to the studied phenotypes and sensitivity methods, found evidence for causal effects of smoking initiation on cannabis initiation and cannabis dependence. In the other direction, they found evidence that cannabis initiation leads to smoking initiation, opioid dependence, and more alcohol consumption. The authors caution that these latter findings may indicate there is shared vulnerability rather than causality, because smoking and alcohol use typically begin before the use of the other substances (the temporality is unlikely) [119]. These findings emphasise that genetic variants for cannabis use, initiation specifically, are pleiotropic and likely not very specific in their effects.

The second focal point in MR literature is the relationship between cannabis use and mental health disorders. A recent systematic review paper summarised all MR studies that looked at substance use and mental health, including eight studies on cannabis use [120]. For major depression, self-harm behaviour, and cognitive functioning (*N* = 126,291 [121], 125,925 [106], and 3242 [122], respectively) there was no clear evidence for causal effects with cannabis initiation, in either direction. Note that the sample size of the analyses looking at cognitive functioning were underpowered. Between liability to schizophrenia and cannabis initiation there was evidence for bidirectional effects, based on three studies that used (partly) overlapping GWAS datasets (*N* = 79,845 [123], 32,330 to 150,064 [124], and 150,064 to 184,765 respectively [75]). Finally, based on two studies there was evidence that liability to ADHD increases the risk of cannabis initiation, without clear evidence for the reverse (*N* = 32,330 to 53,293 [125] and 53,293 to 184,765 [126]). Since this systematic review, other MR studies focussed on cannabis use have been published. One study found evidence that liability to bipolar disorder causally increases the risk of cannabis initiation, but no evidence for the reverse (*N* = 62,082 to 198,882; [127]). A second study found evidence that cannabis initiation causally increases the risk of suicide attempt (*N* = 50,264 to 162,082; [128]), while another found no evidence for causality between cannabis dependence and suicide-related behaviours (*N* = 18,223 to 117,733; [129]). Finally, a particularly comprehensive study investigated cannabis dependence and schizophrenia using multiple causally informative methods (genomic SEM, latent causal variable modelling, and MR) (*N* = 161,405 to 357,806; [130]). Some support was found for a causal influence of cannabis dependence on schizophrenia, but findings were not consistent across methods This last study is a nice demonstration of the importance of using several different methods to study (causal) relationships. This is referred to as ‘triangulation’, the premise being that if methods with different strengths and weaknesses point in the same direction, it is less likely a finding is an artefact [131]. Besides genetic methods, it is important for future studies to triangulate with alternative methods, such as longitudinal epidemiological analyses, or other types of (non-genetic) instrumental variable methods (e.g. population effects of cannabis policy changes).

## Interplay between genetic vulnerability and environmental factors

Both genetic and environmental factors play a role in cannabis use. A complex interplay between these factors might determine individual differences in cannabis use and dependence. Interplay can occur as gene-environment interaction (G × E) where the effect of genetic vulnerability depends on the presence of environmental factors. For example, increased genetic risk for cannabis use may only influence patterns of use in people living in a neighbourhood where cannabis is widely available. Alternatively, genetic effects may reflect gene-environment correlations (rGE), where genetic liability to cannabis use influences environments to which individuals are either exposed or self-select into. For example, having an outgoing personality might lead to exposure to an environment where the use of cannabis is more common. Similarly, genetic effects could influence ones’ socio-economic status and thereby become correlated with one’s social surroundings and geographic location [132].

Rather than relying on candidate genes, G × E interaction studies now typically use polygenic measures [133]. A review of G × E studies using PGSs for substance use outcomes identified 34 publications (publication date before February 2018) but only three studies included cannabis outcome measures, and none used a cannabis use PGS. But since then, five studies have been published using a cannabis PGS to explore G × E interaction (Table 2). Two studies found significant PGS x Environment interactions; for trauma exposure [102] and for community activities [108]. Trauma seemed to exacerbate genetic risk for substance use, while engagement in community activities may serve as protective factor for cannabis use. Other environmental factors such as frequency of religious service attendance, organised sports, school activities, church activities and peer deviance were not or not consistently significant in these studies. The three other studies (exploring moderating roles for neighbourhood environment [134], peer cannabis use [101], prenatal stress, warm parenting, and cortisol reactivity [135]) did not find G × E interactions for cannabis use outcomes.

**Table 2:** Overview of studies exploring gene-environment interaction for cannabis use using polygenic scores.

Study	*N*/*M*_age_	PGS phenotype	Environmental factor/moderator	Outcome measure(s)	Findings
Johnson et al. [101]	1167 (12–26 years)	Cannabis initiation	–peer cannabis use	–trajectory membership (no/low cannabis use, high cannabis use, moderate cannabis use trajectories)	–PRS-by-peer use interaction was not significantly associated with trajectory membership.
Marceau et al. [135]	*N* = 1632, cannabis data used from wave at age 16.	Cannabis initiation	–prenatal stress –warm parenting - cortisol reactivity	–cannabis any use and frequency at age 16	–No interactions between PGS and moderators for cannabis outcomes
Meyers et al. [102]	*N* = 7591 European ancestry, *M*_age_ = 36.8, *N* = 3359 African ancestry, *M*_age_ = 32.9	Cannabis initiation	–trauma exposure; –frequency of religious service attendance	–Cannabis use; –DSM-5 CUD symptom count	–PGS only influenced cannabis use among those exposed to trauma (compared to unexposed). –PRS had a greater influence on cannabis ever use and DSM-5 CUD symptom count among those who less frequently attended religious services as compared to those who more frequently attended services (note: no significant moderations if cross-terms for all variables were added to the models).
Pasman et al. [134]	*N* = 5676, *M*_age_ = 45.3	Cannabis initiation	Neighbourhood environment: –metropolitan factor; –SES factor	–Cannabis use	–No G × E effects, no gene-environment correlation.
Thomas et al. [108]	*N* = 750 European ancestry, *M*_age_ 19.0, *N* = 405 African American ancestry, *M*_age_ = 18.9	Cannabis initiation	–organised sports –school activities –community activities –church activities –peer deviance	–recent cannabis use (yes/no)	–Engagement with community activities moderated the influence of the polygenic risk score in the EA sample, such that PRS was associated with recent cannabis use among those who never engaged in community activities. Not replicated in AA sample. –Engagement with recreational sports also moderated the influence of the PRS in the EA sample, such that there was a significant association between PRS and recent cannabis use among those who frequently engage with recreational sports; however, this effect was no longer significant after multiple testing correction.

*N* = total sample size, *M*_age_ = Mean age of the sample; PGS phenotype = the phenotype the polygenic score is based on; Environmental factor/moderator = the environmental factor or moderator that is used in the study; outcome measure(s) = the cannabis-related outcome measure; Findings = short summary of the main findings (for cannabis).

Regarding rGE, Johnson et al. [101] showed that individuals with high cannabis PGS are more likely to affiliate with cannabis using peers, a finding that is consistent with a process of social selection, whereby higher genetic risks for cannabis use may drive the propensity to affiliate with deviant drug using peers [136]. To our knowledge, only Pasman et al. (2019) have explicitly simultaneously modelled rGE (which was found to be absent) independently from G × E. Although G × E and rGE are typically studied independently, several statistical and conceptual reasons warrant joint assessment [137]. The presence of rGE may lead to false conclusions of G × E as many environmental factors are in fact influenced by genes themselves [137, 138].

In summary, evidence for G × E interactions for cannabis use is limited. Significant interaction need to be replicated and all studies used PGSs for cannabis initiation (based on [75]). Future studies should also evaluate G × E for more severe cannabis measures, but discovery GWAS samples for these phenotypes are still relatively small [79]. Furthermore, other environmental factors need to examined (for example parental factors) and the potential influence of rGE on G × E findings needs to be considered.

## Clinical use

GWAS findings, the identification of mechanistic pathways, and studies investigating PGSs for cannabis use raise questions regarding the predictive validity of cannabis PGSs in clinical settings. Yanes et al. [139] have argued, broadly, that PGSs can be useful in terms of informing population screening programs, guiding therapeutic interventions, refining risk for individuals and families at high risk, and improving diagnosis. To date however, most cannabis research has been limited to basic science studies. While it is viable with PGSs to predict cannabis use in independent target samples, it is important to realise that PGSs currently contain too much noise and explain very little variation (up to a few percent), commensurate with other complex traits. Savatore et al. [140] have illustrated that although PGSs could be used to predict individuals and families meeting fewer clinical criteria for substance use disorders including cannabis, the effect sizes remain very small. Therefore, use of genetic results to identify individuals at risk of substance use disorders is modest at best, and future success depends upon increased and well phenotyped and genotyped samples [141]. It is not possible yet to use PGSs in clinical settings to meaningfully predict an individual’s genetic vulnerability to cannabis use. Efforts by the ICC and PGC-SUD workgroup to ascertain larger samples to improve the predictive validity of cannabis-based PGSs are ongoing. Furthermore, the modest heritability and importance of environmental risks shown by twin studies, suggests that clinical prediction algorithms will likely require a combination of measured genotypes and environments.

## Future directions and conclusions

Insights into the genetic architecture of cannabis use are improving, but there are several steps we need to take in order to learn more [142]. Firstly, increasingly larger GWAS samples are required to capture more heritability. The genome coverage of GWASs also needs to improve to capture rare variants and other types of variation not captured by the current micro-arrays. Furthermore, we need to focus on including individuals of non-European ancestry. GWASs have been done almost exclusively in datasets of European ancestry. Systematic differences in ancestral genetic and environmental influences renders PGSs less useful in non-European samples. We need to improve the coverage of the population (e.g. non-European ancestry) to decrease the effect of ascertainment bias on the genetic signal. Environmental effects need to be accounted for in these genetic association studies by including within family and within region analyses while the interplay between genes and environment should be addressed more thoroughly. Twin studies show that genetic influences are more pronounced for cannabis dependence compared to initiation of cannabis use with similar SNP-based heritabilities from GWASs. Lastly, post-GWAS methodology needs to be further improved in order to disentangle the polygenic effects into underlying traits and underlying biological processes [142].

A specific point of focus in post-GWAS methodology is the improvement of MR and other causal inference methods. So far, the number of genetic variants associated with cannabis use—which are needed to use as instruments in an MR study—is limited. This may lead to weak instrument bias and spurious MR findings. In future studies, it is therefore recommended that evidence from a range of different MR methods is triangulated. Besides correcting for weak instrument bias [143], MR methods that allow correction for ‘correlated horizontal pleiotropy’ are important [144]. This phenomenon—whereby genetic variants affect two traits through a shared heritable factor—is highly relevant when testing relationships between cannabis use and mental health outcomes, but it is not taken into account in most common MR methods. Another promising approach is the MR direction of causation (MR-DoC) model, an adaptation which integrates the twin model with the MR design (a limitation is that well-powered twin samples are required) [145]. Besides genetic methods, it is important for future studies to also triangulate with alternative methods, such as longitudinal epidemiological analyses, or other types of (non-genetic) instrumental variable methods.

In conclusion, human genetics studies have provided a lot of insights of the genetic architecture of cannabis use. A large body of twin studies has shown that cannabis use is heritable—with moderate heritability for initiation of use and a somewhat higher heritability for measures of frequency and CUD. In the past decade, our insights into the molecular genetic architecture of cannabis use has also improved. Increases in sample size and technological advances have enabled GWASs to identify specific locations in the genome that are associated with cannabis use. So far, dozens of genetic variants and genes implicated in cannabis use have been reported, each explaining a tiny fraction of variance. Using summary-level GWAS data also provided us insight into the comorbidity between cannabis use and the use of other substance and mental health problems, providing evidence for shared genetic influences as well as some causal associations.

Future studies with increased sample sizes, including more diverse populations, higher genome coverage, and new approaches to improve the specificity of the genetic signals, should further increase our knowledge of the biological underpinnings of cannabis use and the predictive power of genetics.

## Supplementary information

Supplementary Information
