# Supplementary material for: The genetic aetiology of cannabis use: from twin models to genome-wide association studies and beyond
Source: Transl Psychiatry. 2022 Nov 21;12:489. doi: 10.1038/s41398-022-02215-2 (PMC9678872; doi:10.1038/s41398-022-02215-2)
Supplement: Supplementary file 1 — Supplementary Information [file 41398_2022_2215_MOESM1_ESM.pdf]

Supplementary Information

The genetic etiology of cannabis use: from twin models to genome-wide association studies and beyond.

Karin J.H. Verweij, Jacqueline M. Vink, Abdel Abdellaoui, Nathan A. Gillespie, Eske M. Derks, & Jorien L. Treur

Supplementary Table 1: GWASs and their sample sizes of traits included in the genetic correlation estimations in Figure 2.

Trait	N_{total}	N_{cases}	$N_{controls}$	$N_{effective}$
**Psychiatric disorders:**				
ADHD[1]	55,374	20,183	35,191	51,306
PTSD[2]	206,655	32,428	174,227	109,358
Schizophrenia[3]	130,644	53,386	77,258	126,282
Anxiety[4]	224,330	34,189	190,141	115914
Major depression[5]	431,394	116,404	314,990	161,090
Bipolar disorder[6]	63,766	11,974	51,792	38,902
Autism spectrum disorder[7]	46,350	18,381	27,969	44,367
OCD[8]	576,257	14,140	562,117	55,172
Anorexia[9]	72,517	16,992	55,525	52,042
**Substance use:**				
Cocaine dependence[10]	6,378	2,085	4,293	5614
Alcohol dependence[11]	46,568	11,569	34,999	34,779.54
Smoking initiation[12]	499,000	-	-	318,482
Nicotine dependence[13]	58,000	-	-	58,000
Alcoholic drinks per week[12]	399,604	-	-	262,462
Cigarettes per day[12]	180,438	-	-	112,757
Caffeine[14]	91,462	-	-	91,462
Alcohol use (AUDIT)[15]	121,604	-	-	121,604
Smoking cessation[12]	240,037	-	-	149,155
Age at smoking initiation[12]	181,780	-	-	114,119
**Personality:**				
Risk Taking[16]	466,571	-	-	466,571
Extraversion[17]	29,501	-	-	29,501
Neuroticism[18]	390,278	-	-	390,278
**Cognition & SES:**				
Townsend[19]	112,151	-	-	112,151
Adult IQ[20]	78,308	-	-	78,308
Educational attainment[21]	245,621	-	-	245,621
Household income[22]	505,541	-	-	505,541

N.B. Effective sample size was provided by the authors of the GWASs or calculated with the following formula: $4/(1/N_{cases}+1/N_{controls})$.

References

1 Demontis, D. & Walters, R. K. Discovery of the first genome-wide significant risk loci for attention deficit/hyperactivity disorder. *Nature genetics* **51**, 63-75, doi:10.1038/s41588-018-0269-7 (2019).

2 Nievergelt, C. M. *et al.* International meta-analysis of PTSD genome-wide association studies identifies sex-and ancestry-specific genetic risk loci. *Nature communications* **10**, 1-16 (2019).

3 Trubetskoy, V. *et al.* Mapping genomic loci implicates genes and synaptic biology in schizophrenia. *Nature* **604**, 502-508, doi:10.1038/s41586-022-04434-5 (2022).

4 Levey, D. F. *et al.* Reproducible genetic risk loci for anxiety: results from∼ 200,000 participants in the Million Veteran Program. *American Journal of Psychiatry* **177**, 223-232 (2020).

5 Wray, N. R. *et al.* Genome-wide association analyses identify 44 risk variants and refine the genetic architecture of major depression. **50**, 668-681, doi:10.1038/s41588-018-0090-3 (2018).

6 Mullins, N. *et al.* Genome-wide association study of more than 40,000 bipolar disorder cases provides new insights into the underlying biology. *Nature genetics* **53**, 817-829 (2021).

7 Meta-analysis of GWAS of over 16,000 individuals with autism spectrum disorder highlights a novel locus at 10q24. 32 and a significant overlap with schizophrenia. *Molecular autism* **8**, 1-17 (2017).

8 Strom, N. I. *et al.* Genome-wide association study identifies new locus associated with OCD. *medRxiv* (2021).

9 Watson, H. J. *et al.* Genome-wide association study identifies eight risk loci and implicates metabo-psychiatric origins for anorexia nervosa. *Nature genetics* **51**, 1207-1214 (2019).

10 Cabana-Domínguez, J., Shivalikanjli, A., Fernàndez-Castillo, N. & Cormand, B. Genome-wide association meta-analysis of cocaine dependence: Shared genetics with comorbid conditions. *Progress in Neuro-Psychopharmacology and Biological Psychiatry* **94**, 109667 (2019).

11 Walters, R. K. *et al.* Transancestral GWAS of alcohol dependence reveals common genetic underpinnings with psychiatric disorders. *Nature neuroscience* **21**, 1656-1669 (2018).

12 Liu, M., Jiang, Y. & Wedow, R. Association studies of up to 1.2 million individuals yield new insights into the genetic etiology of tobacco and alcohol use. *Nature genetics* **51**, 237-244, doi:10.1038/s41588-018-0307-5 (2019).

13 Quach, B. C. *et al.* Expanding the genetic architecture of nicotine dependence and its shared genetics with multiple traits. *Nature communications* **11**, 1-13 (2020).

14 Cornelis, M. C. *et al.* Genome-wide meta-analysis identifies six novel loci associated with habitual coffee consumption. *Molecular psychiatry* **20**, 647-656, doi:10.1038/mp.2014.107 (2015).

15 Sanchez-Roige, S. *et al.* Genome-wide association study meta-analysis of the Alcohol Use Disorders Identification Test (AUDIT) in two population-based cohorts. *American Journal of Psychiatry* **176**, 107-118 (2019).

16 Linnér, R. K. *et al.* Genome-wide association analyses of risk tolerance and risky behaviors in over 1 million individuals identify hundreds of loci and shared genetic influences. *Nature genetics* **51**, 245-257 (2019).

17 van den Berg, S. M. *et al.* Meta-analysis of Genome-Wide Association Studies for Extraversion: Findings from the Genetics of Personality Consortium. *Behav Genet* **46**, 170-182, doi:10.1007/s10519-015-9735-5 (2016).

18 Nagel, M. *et al.* Meta-analysis of genome-wide association studies for neuroticism in 449,484 individuals identifies novel genetic loci and pathways. *Nature genetics* **50**, 920-927 (2018).

19 Hill, W. D. *et al.* Molecular genetic contributions to social deprivation and household income in UK Biobank. *Current Biology* **26**, 3083-3089 (2016).

20 Sniekers, S. *et al.* Genome-wide association meta-analysis of 78,308 individuals identifies new loci and genes influencing human intelligence. *Nature genetics* **49**, 1107 (2017).

21 Lee, J. J. *et al.* Gene discovery and polygenic prediction from a 1.1-million-person GWAS of educational attainment. *Nature genetics* **50**, 1112 (2018).
22 Hill, W. D. *et al.* Genome-wide analysis identifies molecular systems and 149 genetic loci associated with income. *Nature communications* **10**, 5741, doi:10.1038/s41467-019-13585-5 (2019).
